# ORF10–Cullin-2–ZYG11B complex is not required for SARS-CoV-2 infection

**DOI:** 10.1073/pnas.2023157118

**Published:** 2021-04-07

**Authors:** Elijah L. Mena, Callie J. Donahue, Laura Pontano Vaites, Jie Li, Gergely Rona, Colin O’Leary, Luca Lignitto, Bearach Miwatani-Minter, Joao A. Paulo, Avantika Dhabaria, Beatrix Ueberheide, Steven P. Gygi, Michele Pagano, J. Wade Harper, Robert A. Davey, Stephen J. Elledge

**Affiliations:** ^a^Division of Genetics, Department of Genetics, Brigham and Women’s Hospital, Harvard Medical School, Boston, MA 02115;; ^b^Howard Hughes Medical Institute, Brigham and Women's Hospital, Boston, MA 02115;; ^c^Department of Microbiology, National Emerging Infectious Disease Laboratories, Boston University Medical Campus, Boston, MA 02118;; ^d^Department of Cell Biology, Blavatnik Institute, Harvard Medical School, Boston, MA 02115;; ^e^Department of Biochemistry and Molecular Pharmacology, New York University Grossman School of Medicine, New York, NY 10016;; ^f^Howard Hughes Medical Institute, New York University Grossman School of Medicine, New York, NY 10016;; ^g^Proteomics Laboratory, Division of Advanced Research Technologies, New York University Grossman School of Medicine, New York, NY 10016;; ^h^Department of Neurology, New York University Grossman School of Medicine, New York, NY 10016

**Keywords:** SARS-CoV-2, ORF10, ZYG11B, ZER1, CUL2

## Abstract

In order to understand the transmission and virulence of severe acute respiratory syndrome coronavirus 2 (SARS-CoV-2), it is necessary to understand the functions of each of the gene products encoded in the viral genome. One feature of the SARS-CoV-2 genome that is not present in related, common coronaviruses is ORF10, a putative 38-amino acid protein-coding gene. Proteomic studies found that ORF10 binds to an E3 ubiquitin ligase containing Cullin-2, Rbx1, Elongin B, Elongin C, and ZYG11B (CRL2^ZYG11B^). Since CRL2^ZYG11B^ mediates protein degradation, one possible role for ORF10 is to “hijack” CRL2^ZYG11B^ in order to target cellular, antiviral proteins for ubiquitylation and subsequent proteasomal degradation. Here, we investigated whether ORF10 hijacks CRL2^ZYG11B^ or functions in other ways, for example, as an inhibitor or substrate of CRL2^ZYG11B^. While we confirm the ORF10−ZYG11B interaction and show that the N terminus of ORF10 is critical for it, we find no evidence that ORF10 is functioning to inhibit or hijack CRL2^ZYG11B^. Furthermore, ZYG11B and its paralog ZER1 are dispensable for SARS-CoV-2 infection in cultured cells. We conclude that the interaction between ORF10 and CRL2^ZYG11B^ is not relevant for SARS-CoV-2 infection in vitro.

## Significance

Understanding the functions of the genes encoded in the SARS-CoV-2 genome is imperative to understanding its pathogenesis. One unique feature of the SARS-CoV-2 genome is ORF10, a small putative protein that was hypothesized to promote infection by hijacking a cellular E3 ubiquitin ligase, CRL2^ZYG11B^. Here, we investigate whether ORF10 hijacks CRL2^ZYG11B^ or functions in other ways, such as to inhibit CRL2^ZYG11B^ or be degraded by it. We do not find evidence that ORF10 regulates or is regulated by CRL2^ZYG11B^, and, furthermore, we find that ZYG11B and its paralog are dispensable for SARS-CoV-2 infection in cultured cells.

Severe acute respiratory syndrome coronavirus 2 (SARS-CoV-2) and the associated COVID-19 pandemic has led to over 30,000,000 infections and 550,000 deaths in the United States alone, as of April 2021 (1). In an effort to understand the molecular basis for the virulence and infectivity of SARS-CoV-2, proteomic experiments have been performed on viral SARS-CoV-2 proteins in order to identify host processes that are regulated or usurped by SARS-CoV-2 (2–4). These studies have identified novel viral−host interactions. Some of these host factors can be modulated by existing drugs, and these drugs have been suggested as possible therapeutics in the treatment of COVID-19 (2).

A proteomic survey of SARS-CoV-2 viral proteins showed that ORF10, a putative 38-amino acid viral protein encoded in the 3′ accessory region of the genome, bound to components of a Cullin-2−RING−ligase (CRL2) complex containing Cullin-2, RBX1, Elongin B, Elongin C, and ZYG11B (CRL2^ZYG11B^) (2). CRL2^ZYG11B^ functions as a ubiquitin ligase (E3), a class of enzymes that attach one or more ubiquitin molecules onto substrate proteins, targeting them for degradation by the proteasome. The CRL2^ZYG11B^ ubiquitin ligase and its paralog CRL2^ZER1^ have been previously shown to function in the N-end degron pathway, where they degrade proteins bearing methionine−glycine−psi, where psi is a bulky or aromatic residue (5). Intriguingly, the N terminus of ORF10 begins with a methionine−glycine−tyrosine motif, which would presumably allow for ORF10 to be recruited to this CRL2^ZYG11B^ ubiquitin ligase complex.

We and others (2) reasoned that ORF10 may function to promote the infection or virulence of SARS-CoV-2 through its interaction with CRL2^ZYG11B^. In this study, we considered three different scenarios for this interaction. First, ORF10 may be a substrate of CRL2^ZYG11B^ given that it contains the preferred N-end rule motif and is subsequently targeted for degradation. Second, ORF10 may bind and inhibit ZYG11B. Since ORF10 is small and contains no lysines, it may be able bind to the substrate binding site of ZYG11B without getting ubiquitylated itself and inhibit CRL2^ZYG11B^ by occluding the binding of its substrates. Third, ORF10 may hijack ZYG11B for the purposes of recruiting new substrates whose degradation is advantageous to the virus. We note that these scenarios are not necessarily mutually exclusive.

The possibility that ORF10 binds to ZYG11B and hijacks it for the purposes of degrading other proteins is particularly interesting. A hijacking scenario would allow ORF10 to impact viral replication by recruiting host antiviral protein(s) to the CRL2^ZYG11B^ complex for ubiquitylation and eventual degradation. Ubiquitin ligase hijackers need not be large, since they typically do not encode any enzymatic activity themselves, but instead tether proteins to host E3 ubiquitin ligases. The potential role of the CRL2^ZYG11B^−ORF10 complex during infection has led one group to propose Pevonedistat (MLN4924), an inhibitor of CRL activity, as a possible therapeutic for treating COVID-19 (2).

Viral hijacking of host ubiquitin ligases has been described in numerous viral infections. For instance, the RNA virus HIV-1 encodes VPU, VPR, and VIF which each hijack different Cullin−RING−ligase complexes in order to degrade host antiviral proteins and thereby promote infection (6, 7). Additionally, the hepatitis B protein HBX hijacks a Cullin-4–containing E3 ubiquitin ligase complex (8), while adenovirus E4(orf6) hijacks a Cullin-2 complex (9).

Here, we validated the interaction between ORF10 and components of the CRL2^ZYG11B^ ubiquitin ligase complex. We do not find any evidence, however, that ORF10 is strongly regulated by CRL2^ZYG11B^ or that it inhibits CRL2^ZYG11B^ activity. Importantly, we also find that cells deficient in ZYG11B and ZER1 still propagate SARS-CoV-2 equally well in vitro, suggesting that the ORF10−ZYG11B interaction is not relevant for SARS-CoV-2 infection.

## Results

### ORF10 Forms a Complex with CRL2-ZYG11B.

**Fig. 1.**
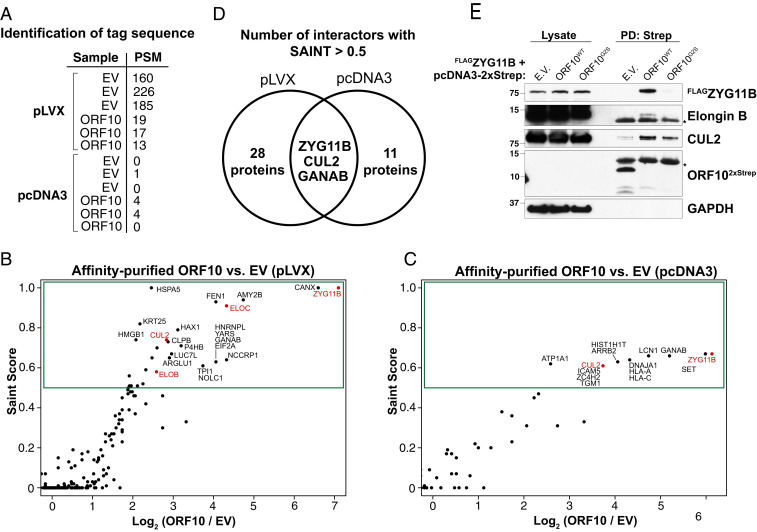
ORF10 forms a complex with CRL2-ZYG11B. (*A*) ORF10 fusion is purified. Table shows the number of PSMs of the tag sequence (GGGSGGGSGGGSWSHPQFEK) detected in each technical replicate. (*B*) An ORF10−2xStrep fusion in the pLVX vector was expressed in 293T cells and affinity purified. ORF10 and copurifying proteins were eluted from streptactin resin using Laemmli sample buffer, and proteins in three technical replicates were quantified using MudPIT mass spectrometry relative to empty vector controls. SAINT scores are a measure of the statistical significance of proteins. Components of the CRL2^ZYG11B^ complex are in red. (*C*) As in *B*, except that ORF10−2xStrep was expressed from a pcDNA3 vector, and purified proteins were eluted using biotin. (*D*) ZYG11B is a reproducible, specific interactor of ORF10. The number of hits in each experiment using a SAINT score cutoff of 0.5 and the overlapping proteins are shown with a Venn diagram. (*E*) ORF10−2xStrep or empty vector (E.V.) control was expressed with FLAG-ZYG11B. Cellular lysates and Streptactin-bound precipitates were immunoblotted as indicated. Asterisks denote nonspecific bands.

To investigate the function of ORF10, we affinity purified ORF10 and identified interacting proteins by mass spectrometry. First, we used a C-terminal 2xStrep tagged version of ORF10, as used previously (2). In these experiments, we were unable to directly detect ORF10, likely owing to its small size and few tryptic peptides, but we were able to detect the affinity tag that it was fused to, suggesting that it was expressed and purified (Fig. 1*A*). We performed two experiments with different vector backbones and protein elution strategies. The first experiment used a pLVX backbone, and proteins were eluted with Laemmli sample loading buffer (Fig. 1*B* and Dataset S1), while the second experiment used a plasmid cloning DNA 3 (pcDNA3) vector, and proteins were eluted using biotin (Fig. 1*C* and Dataset S2). Each experiment included three technical replicates with ORF10 and three with the empty vector control expressing the tag only. Spectral counts of each protein were compared against 14 control affinity purifications in order to calculate the significance analysis of interactome (SAINT) score (10, 11).

In both experiments, we identified ZYG11B among the highest-scoring hits. CUL2 also scored in both experiments, while Elongin B and Elongin C scored as hits in the pLVX experiment. Elongin B, Elongin C, Cullin-2, and RBX1/2 are all known to function with ZYG11B in the CRL2^ZYG11B^ complex (5, 12). Using a SAINT score cutoff of 0.5, three proteins—ZYG11B, CUL2, and GANAB—were found as hits in both replicates (Fig. 1*D*). Since GANAB was a common contaminant in the CRAPome database (13), we focused our attention on the interaction between ORF10 and the E3 ubiquitin ligase CRL2^ZYG11B^.

We confirmed the interaction of ORF10 with CRL2^ZYG11B^ by purifying ORF10^2xStrep^ followed by immunoblotting. Despite the poor expression of ORF10, it specifically bound ^FLAG^ZYG11B (Fig. 1*E*). We also detected the interaction of ORF10 with the endogenous CRL2 proteins CUL2 and Elongin B. Here, we used ORF10^G2S^ as a negative control, because the N-terminal Gly to Ser mutation has been shown, for characterized CRL2^ZYG11B^ substrates, to abolish their binding and degradation (5). Therefore, the N terminus of ORF10—like canonical substrates—is critical for binding to CRL2^ZYG11B^.

### Effect of ORF10 on the Proteome.

Since we confirmed that ZYG11B bound to ORF10, this complex could be altering the cellular proteome in a manner to promote SARS-CoV-2 infection. Alterations to the proteome could be caused by inhibition, activation, or hijacking of CRL2^ZYG11B^.

**Fig. 2.**
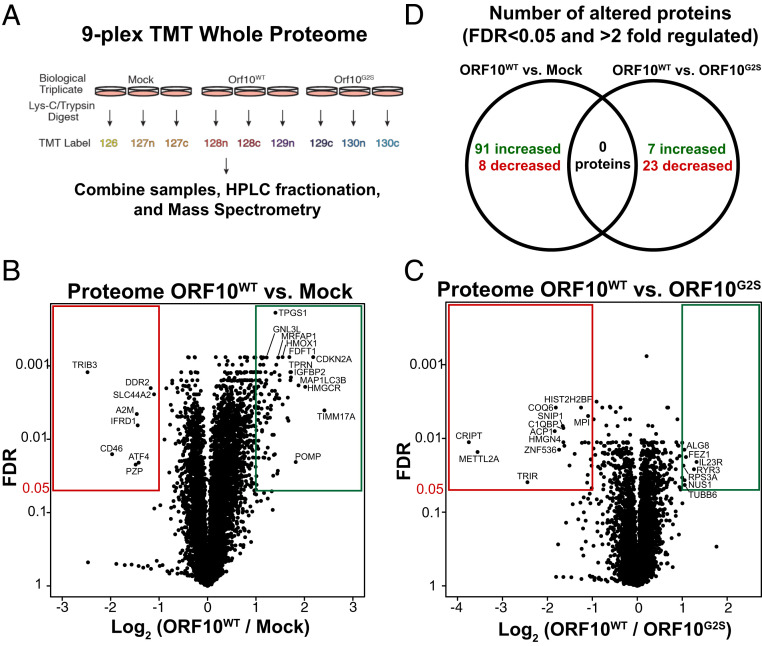
Proteomic changes induced by ORF10 expression. (*A*) The nine-plex TMT approach for quantifying changes to the proteome induced by ORF10. (*B*) Changes to the proteome induced in ORF10^WT^-expressing cells versus the parental 293T cells. (*C*) Changes to the proteome induced in ORF10^WT^-expressing cells versus the ORF10^G2S^-expressing cells. (*D*) No significant changes to the proteome are seen in ORF10^WT^ cells relative to parental cells that are also altered relative to ORF10^G2S^ cells.

In order to characterize changes caused by ORF10 in a systematic manner, we performed a quantitative whole proteome analysis of parental 293T cells and cells stably expressing ORF10^WT^ or ORF10^G2S^. Using a nine-plex tandem mass tag (TMT) strategy (Fig. 2*A*), we obtained consistent labeling and were able to identify 8,195 proteins in the proteome (Fig. 2*B* and Dataset S3). Using a 5% false discovery rate (FDR) cutoff, we observed eight proteins that were down-regulated more than twofold and 91 proteins that were up-regulated more than twofold in ORF10^WT^ cells versus mock 293T cells (Fig. 2*B*).

There were 30 proteins differentially expressed between ORF10^WT^ and ORF10^G2S^ cells. Of these, 7 proteins were increased and 23 were significantly decreased in ORF10^WT^ cells, including CRIPT and METTL2A (Fig. 2*C*). These 30 regulated proteins, however, were not regulated in a ORF10-dependent manner, as they were similarly abundant in cells without ORF10 as in ORF10^WT^ cells (Fig. 2*B*). None of these regulated proteins were enriched in the ORF10 affinity-purified samples (Fig. 1). While we detect some changes induced by ORF10, we failed to identify either proteins that are specifically regulated by wild-type ORF10 or proteins that are also found in association with ORF10 which would suggest a possible role of ORF10 in recruiting those proteins for degradation.

### ORF10 Regulation of CRL2-ZYG11B Activity.

**Fig. 3.**
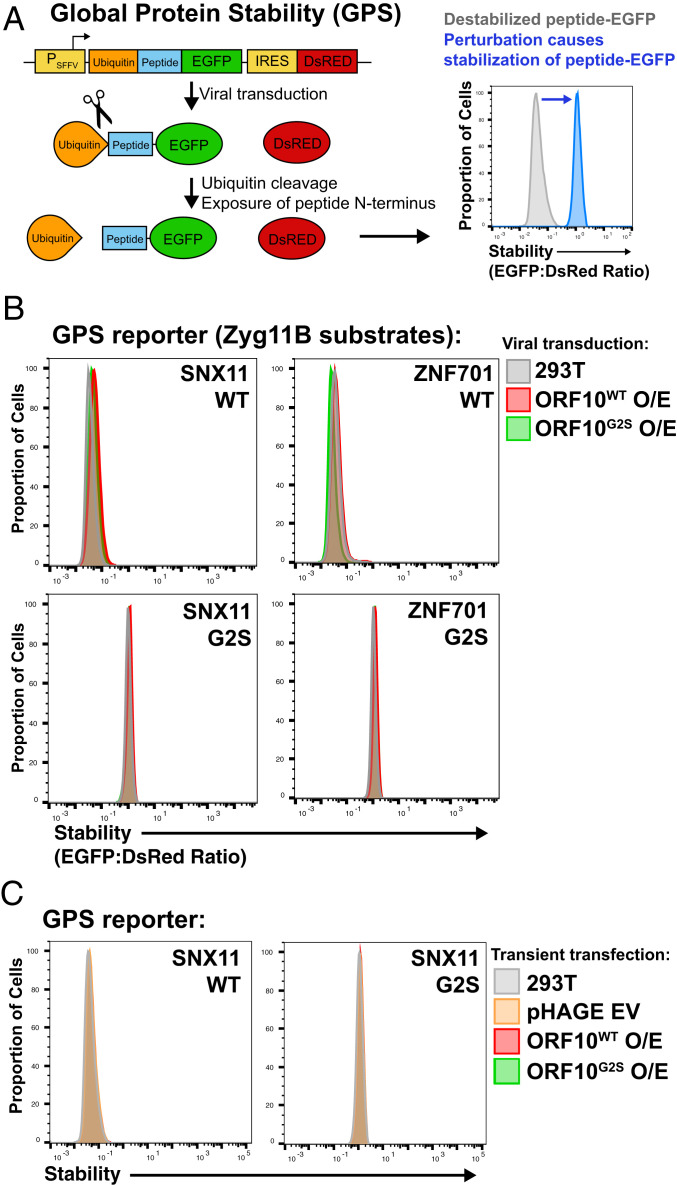
ORF10 does not inhibit CRL2-ZYG11B activity. (*A*) The GPS assay can be used to assess the stability of proteasome substrates by monitoring the EGFP to DsRed ratio of cells expressing the cassette. (*B*) ORF10 does not inhibit ZYG11B or ZER1 activity. Characterized GPS substrates of CRL2-ZYG11B and CRL2-ZER1, SNX11 and ZNF701, were lentivirally expressed together with ORF10. No stabilization of ZYG11B/ZER1 reporters was observed in the presence of ORF10 expression. The G2S mutants of the GPS substrates and of ORF10 are negative controls. (*C*) As in *B*, except ORF10 was transiently transfected using Polyjet.

We further investigated the possibility that ORF10 binds CRL2^ZYG11B^ to inhibit its activity. An inhibitor of CRL2^ZYG11B^ could help promote SARS-CoV-2 infection if CRL2^ZYG11B^ functions to antagonize the virus. To investigate this hypothesis, we used the Global Protein Stability (GPS) system (Fig. 3*A*) (14, 15) with reporters for CRL2^ZYG11B^ and CRL2^ZER1^ activity. In this system, a lentivirally integrated transgene expresses a peptide fused to EGFP and also contains an internal ribosome entry site (IRES) that drives DsRed expression. DsRed is an important component of GPS, since it controls for most perturbations that would affect transcription or translation generally. The amount of peptide−EGFP fusion that is expressed, relative to the DsRed control, is a reliable metric for the stability of the EGFP fusion. Since CRL2^ZYG11B^ recognizes N-terminal degrons, we used GPS constructs that were used previously and that allow us to precisely control the N terminus. These constructs contain a ubiquitin moiety fused to the N terminus of the peptide which is rapidly cleaved following translation to reveal the native N terminus of the peptide.

Our assay used GPS reporters containing N-terminal peptides of ZNF701 and SNX11, previously characterized substrates of CRL2^ZYG11B^ and CRL2^ZER1^. As negative controls, we used the G2S mutants of these peptides that are not recognized by the ubiquitin ligases. We reasoned that, if ORF10 inhibited the functions of CRL2^ZYG11B^ and CRL2^ZER1^, then the GFP:DsRed ratio would increase as fewer EGFP fusion molecules would be targeted for degradation. Nonetheless, we found that lentivirally integrated ORF10 did not affect the stability of these GPS reporters (Fig. 3*B*). In these experiments, we used an untagged ORF10 in order to rule out the possibility that an affinity tag might disrupt the function of this small protein.

We considered the possibility that ORF10 was insufficiently expressed to affect CRL2^ZYG11B^ or CRL2^ZER1^ activity, so we also tested the effects of transient expression of ORF10 using a lipid-based transfection reagent. We found that transient expression of ORF10, as with lentiviral expression, did not affect the activity of CRL2^ZYG11B^/CRL2^ZER1^ (Fig. 3*C*). These results show that ORF10 expression does not inhibit CRL2^ZYG11B^ or CRL2^ZER1^.

### ORF10 Is Not a Substrate of ZYG11B and ZER1.

**Fig. 4.**
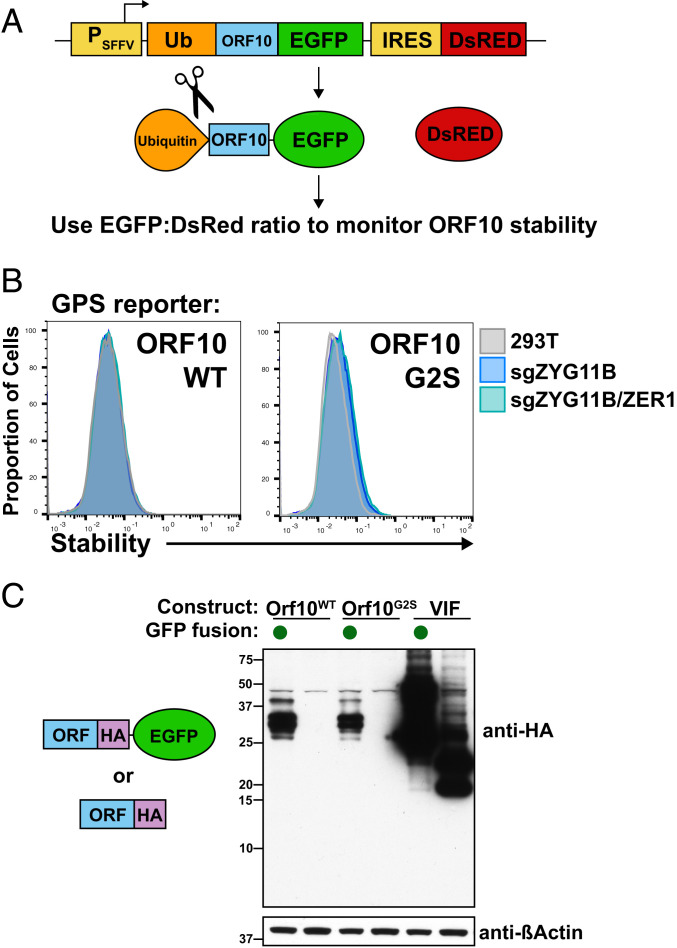
ORF10 is not degraded by CRL2-ZYG11B or CRL2-ZER1. (*A*) ORF10 was fused to EGFP in a GPS cassette in order to assess its stability. We also used an N-terminal ubiquitin fusion, which is quickly cleaved posttranslationally to reveal the endogenous N terminus. (*B*) ORF10 is not stabilized in ZYG11B/ZER1 KO cells. Wild-type 293T, sgZYG11B, or sgZYG11B/ZER1 cells were used to express ORF10 in a GPS fusion. (*C*) The G2S mutant or wild-type form of ORF10 was expressed with or without an EGFP fusion. The fusion to EGFP stabilizes ORF10, whereas the G2S mutant does not stabilize ORF10. VIF is an unrelated control protein from HIV-1.

**Fig. 5.**
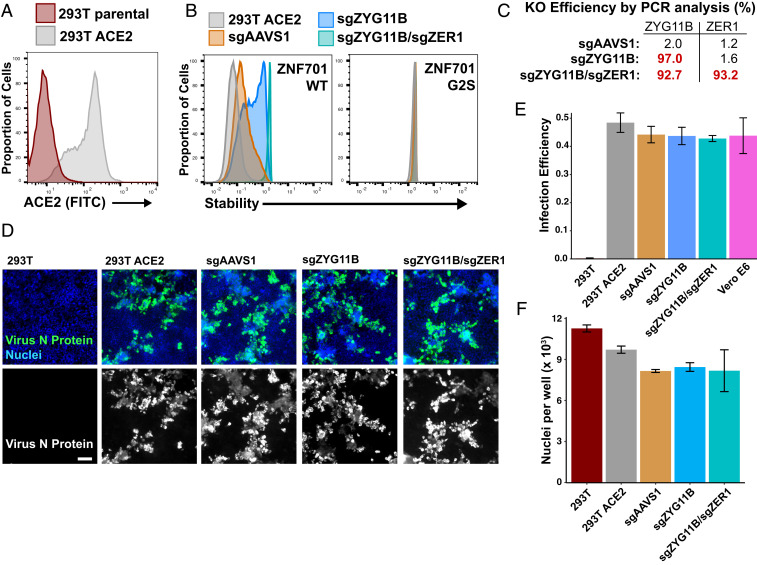
ZYG11B and ZER1 are not required for SARS-CoV-2 infection. (*A*) ACE2 was lentivirally expressed in 293T cells to allow them to be permissible to SARS-CoV-2 infection. ACE2 was detected on the surface of 293T cells by staining with an anti-ACE2 antibody. (*B*) The 293T−ACE2 cells were infected with Cas9 and guides to AAVS1, a control, or to ZYG11B and ZER1. Control and knockout cells were assessed for ZYG11B/ZER1 activity with a GPS assay. (*C*) The genomic regions of ZYG11B and ZER1 were amplified from sgRNA cells, and KO efficiency was determined by TIDE analysis. (*D*) The 293T−ACE2 cells were infected with SARS-CoV-2 and fixed 2 d after infection. SARS-CoV-2 N protein was stained to assess viral infection and replication. (Scale bar, 100 µm.) (*E*) Control and KO cells have similar percentages of infected cells. Error bars depict SD of three replicates. (*F*) Cells were stained with Hoechst, and nuclei were counted. ACE2-expressing cell lines have no significant difference in cell numbers following infection. Error bars depict SD of three replicates.

Since we do not find evidence that ORF10 is acting as an inhibitor or hijacker of CRL2^ZYG11B^, we next tested a scenario in which ORF10 is simply a substrate of CRL2^ZYG11B^ and is being targeted for degradation. In order to test this possibility, we constructed a GPS cassette in which ORF10 was fused to EGFP (Fig. 4*A*). This GPS reporter, when expressed, had a low EGFP-to-DsRed ratio, indicative of instability. The ORF10 GPS construct was not stabilized in sgZYG11B or sgZYG11B/sgZER1 knockout (KO) cells (Fig. 4*B*), in contrast to the characterized substrate ZNF701 (Fig. 5*B*). Furthermore, the G2S construct of ORF10 was not stabilized relative to wild-type ORF10 (Fig. 4 *B* and *C*), further suggesting that ORF10 is not appreciably degraded via CRL2^ZYG11B^ or CRL2^ZER1^.

While these results seem surprising, it is possible that ORF10−GFP evades ubiquitylation by ZYG11B due to folding or steric hindrance by GFP. Alternatively, ORF10 may be predominantly degraded through a different pathway such that ZYG11B and ZER1 may have only a small effect on its overall abundance. The relatively low stability of ORF10 suggests the latter explanation is at least partially responsible.

### ZYG11B and ZER1 Are Not Required for SARS-CoV-2 Infection.

Finally, we tested whether ORF10 may be generally functioning with CRL2^ZYG11B^ or CRL2^ZER1^ in a way that promotes infection. We investigated this possibility by using cells in which ZYG11B, alone or in combination with ZER1, was knocked out. If ORF10 depended on CRL2^ZYG11B^ and/or CRL2^ZER1^ in order to promote infection, then ZYG11B/ZER1 KO cells would be expected to be resistant to SARS-CoV-2 infection.

We were able to use 293T cells for our infection model because we introduced ACE2 and validated the expression of it on the surface of cells (Fig. 5*A*). The introduction of ACE2 makes 293T cells permissible to SARS-CoV-2 (Fig. 5 *D* and *E*), consistent with previous reports (16, 17). We then introduced Cas9 and single guide RNAs (sgRNAs) against AAVS (a negative control guide), ZYG11B, or a combination of ZYG11B and ZER1 guides into the 293T−ACE2 cells. KO cells were validated to be defective in ZYG11B and ZER1 activity using GPS reporters (Fig. 5*B*). Consistent with previous data (5), the ZNF701 reporter is most stabilized in ZYG11B/ZER1 double-KO cells, indicating some redundancy between ZYG11B and ZER1. We also confirmed that the KO efficiency was greater than 90% in all of the cell lines, by genomic amplification and TIDE (tracking of indels by decomposition) analysis (18) (Fig. 5*C*).

Control and KO cells were infected with SARS-CoV-2 at a multiplicity of infection (MOI) of 0.4, which is low enough to allow for multiple rounds of viral replication. Two days postinfection, cells were fixed and stained for DNA and SARS-CoV-2 nucleoprotein (N). Any defects in SARS-CoV-2 infection or replication would be observed as a decrease in N staining. We saw no appreciable differences in protein N staining between control cells and sgZYG11B KO cells or sgZYG11B/sgZER1 double-KO cells (Fig. 5 *D* and *E*). We also observed similar numbers of cells, as quantified by Hoechst staining, among the permissive (ACE2-expressing) cell lines (Fig. 5*F*). These data show that the ORF10−CRL2^ZYG11B^ complex is not required for SARS-CoV-2 infection in vitro.

## Discussion

Here, we validate that ORF10 interacts with the E3 ubiquitin ligase complex composed of CUL2, Elongin B, Elongin C, and ZYG11B. We investigated several possible scenarios in which the interaction of ORF10 with CRL2^ZYG11B^ or the related E3 ubiquitin ligase CRL2^ZER1^ might play a functional role in SARS-CoV-2 infection.

While we observe robust binding of ORF10 to CRL2^ZYG11B^ in a manner that depends on the N-terminal residues of ORF10, no effect on CRL2^ZYG11B^ function was found. First, we do not find any evidence that ORF10 regulates CRL2^ZYG11B^ complex by activating, inhibiting, or hijacking it. Second, proteomic analysis did not yield any obvious degradative substrates of a CRL2^ZYG11B^−ORF10 complex. Third, we find that ORF10 is not strongly degraded via CRL2^ZYG11B^. Fourth, ZYG11B and ZER1 activity is dispensable for SARS-CoV-2 infection in vitro. While we cannot rule out that ORF10 may have other roles that are important for infection, any effect that ORF10 may have on ZYG11B is not relevant for SARS-CoV-2 infection in vitro.

There are four pieces of evidence from the recent literature that suggest that the ORF10 protein may have little functional impact on SARS-CoV-2 infection. First, transcriptome analysis of infected cells found limited or no evidence for a distinct subgenomic messenger RNA (mRNA) containing a 5′ ORF10, in contrast to the other accessory genes which possess their own mRNA (4, 19). Second, while efforts to identify the abundance of ORF10 using mass spectrometry may miss ORF10 because it is small and contains few tryptic peptides (4), ribosome footprinting studies can also report on whether an RNA is translated (20). A recent ribosome footprinting study found that there was a small peak at the start codon of ORF10 in the presence of harringtonine or lactimidomycin, drugs that stall the ribosome. However, the overall ribosome occupancy of ORF10 was lower than for every other canonical accessory gene and was similar in occupancy to the 3′ untranslated region (21). These results suggest that ORF10 is translated less than the other accessory genes. Since we found that ORF10 is not well expressed in many contexts (Fig. 4), these data suggest that the ORF10 protein may only be fleetingly present during infection, if expressed at all. Third, a strain of SARS-CoV-2 in which ORF10 contains a premature stop codon at residue 29 was isolated, and it can still replicate in vivo and in vitro (22). While most of ORF10, including the N terminus used to bind ZYG11B, is still retained in this strain, this nonetheless implies that the full open reading frame is not required for infection. Fourth, while ORF10 is conserved in SARS-CoV-2, the protein coding sequence is not conserved in diverse Sarbecovirus genomes, suggesting that the RNA sequence may play a regulatory role rather than a coding function (23). These results in the literature, together with our findings, should collectively be interpreted in assessing the functional importance of ORF10.

## Materials and Methods

### Cell Culture.

HEK-293T cells (ATCC CRL-3216) were maintained in Dulbecco’s Modified Eagle Medium (DMEM) GlutaMAX (Life Technologies) supplemented 10% fetal bovine serum (GE HyClone) and penicillin/streptomycin (Life Technologies). Cells were periodically assayed with MycoAlert (Lonza) to ensure that they were free of mycoplasma.

### Transfection and Lentiviruses.

Lentiviruses were prepared by transfecting HEK-293T cells with 1 μg of transfer vector and 1 μg of a mixture of plasmids encoding Gag-Pol, Rev, Tat, and VSV-G using Polyjet In Vitro DNA Transfection Reagent (SignaGen Laboratories). Cells were seeded into six-well plates at 1.2 million cells per well the day prior to transfection. The day following transfection, media was changed. Two days following transfection, viral supernatant was harvested, cell debris was removed by centrifugation, and viral aliquots were stored at −80 °C. Cells were transduced with virus in the presence of 10 μg/mL hexadimethrine bromide (Polybrene, Millipore). Two to three days following transduction, cells were selected with puromycin (2 μg/mL, Clontech) or hygromycin (200 μg/mL, ThermoFisher Scientific).

In order to create double-KO cell lines, ACE2-hygro cells were infected with an equal volume of sgZYG11B and sgZER1 lentiCRISPR v2 viruses and selected with puromycin.

Transfection of ORF10 was performed in six-well plates with 1 μg pHAGE−pSFFV−ORF10−PGK−Hygro construct or control vector and 6 μL of Polyjet. Media was changed at 24 h, and cells were analyzed by flow cytometry at 48 h. High (>80%) transfection efficiency was verified by using a GFP control plasmid in a separate well.

### Constructs and Antibodies.

ORF10 was PCR amplified from a SARS-CoV-2 library generated previously (24). To minimize the effects of epitope tags, we used untagged ORF10 when possible. We made pHAGE−pCMV−ORF10−PGK−Puro (Fig. 2*B*) and pHAGE−pSFFV−ORF10−PGK−Hygro (Fig. 2*C*) constructs using Gibson Assembly (NEB). SNX11 and ZNF701 N-terminal degron GPS constructs were reported previously (5). The GPS constructs containing ORF10 and VIF were amplified with a C-terminal HA tag and inserted into pHAGE−pSFFV−Ub−SalI−Linker−EGFP−IRES−DsRed−pPGK−Hygro either in frame with the EGFP or following removal of the EGFP. An entry vector containing human ACE2 from the Ultimate ORF (ThermoFisher Scientific) library was gateway cloned in pHAGE−Trex−DEST−Hygro using LR Clonase II (ThermoFisher Scientific), per the manufacturer’s protocol.

ORF10 tagged with C-terminal twin-STREP (pLVX ORF10−2Strep) was a gift of Nevan Krogan (University of California San Francisco, San Francisco, CA) (Addgene #141394). The pLVX empty vector was generated by removing ORF10. The pcDNA3-2Strep was generated in our laboratory from the empty pcDNA3 backbone. ORF10 complementary DNA was amplified from pLVX ORF10−2Strep and cloned in the pcDNA3-2Strep empty vector to generate the pcDNA3−ORF10−2Strep. The G2S mutant was generated in pcDNA3 ORF10−2xStrep by Quikchange (Agilent). For KO studies, lentiCRISPR v2 constructs were cloned as described (25) using the following guide sequences:

AAVS1, GGG​GCC​ACT​AGG​GAC​AGG​AT
ZYG11B, GCG​CTC​GTA​AGG​ATC​CTC​GA
ZER1, GCC​GCA​GCA​GGG​ACT​CCA​CA.

We used the following antibodies: anti-HA (Cell Signaling, C29F4), anti-beta Actin (Santa Cruz, sc-8432), anti-GAPDH (Cell Signaling, D16H11), anti-FLAG (Sigma, F1804), anti-CUL2 (Santa Cruz, sc-166506), anti-Elongin B (Santa Cruz, sc-133090), anti-Strep tag (IBA Lifesciences, 2-1507-001), anti-ACE2 (R&D Systems, AF933), anti-SARS-CoV-2 nucleoprotein (Sino Biological, 40143-R004), chicken Alexa Fluor 488 anti-goat (Invitrogen, A21467), goat Alexa Fluor 488 anti-rabbit (Invitrogen A11008), HRP-conjugated goat anti-mouse (Thermo Fisher, 31430), and HRP-conjugated goat anti-rabbit (Thermo Fisher, 31460).

KO cells were genotyped by extracting genomic DNA using Gentra Puregene (Qiagen), regions were amplified using Q5 polymerase (NEB) with the following primers, and sequences were analyzed with TIDE, https://tide.nki.nl/ (18):

ZYG11B (forward), TGA​ATG​ATG​GAA​CTG​TGG​G
ZYG11B (forward), TTC​AGA​TGT​TTG​AGT​TCC​CGA
ZER1 (forward), AGG​GTG​GTG​ATG​ATG​ATG​G
ZER1 (reverse), CCT​GAG​GGC​GTG​ACA​ACA.

### Flow Cytometry.

All flow cytometries were acquired on a BD LSRII instrument (Becton Dickinson), and data were analyzed and plotted in FlowJo (v10.7). To verify ACE expression, HEK-293T ACE2 cells were stained with anti-ACE2 antibody in phosphate-buffered saline (PBS) containing 1% bovine serum albumin (BSA) for 1 h, washed again with PBS, stained with Alex Fluor 488 secondary antibody for 30 min, washed twice with PBS, and analyzed.

### ORF10 Affinity Purification/Western Blotting.

HEK-293T cells were transiently transfected using Polyjet (SignaGen). Approximately 48 h after transfection, cells were incubated with Bortezomib (Selleckchem) for 3 h before collection. Cell lysis was carried out with lysis buffer (50 mM Tris pH 8.0, 150 mM NaCl, 10% glycerol, and 0.5% Nonidet P-40) supplemented with protease and phosphatase inhibitors. After centrifugation at 15,000 × *g* for 15 min, lysates were incubated with Streptavidin-conjugated magnetic beads (IBA #2-4090-002). Beads were washed with lysis buffer, and precipitated proteins were eluted with Laemmli sample loading buffer.

Proteins were resolved using a Novex 16% Tricine sodium dodecyl sulfate polyacrylamide gel electrophoresis (SDS/PAGE) gel (Thermo Fisher) with tricine SDS running buffer and transferred to nitrocellulose membranes (TransBlot Turbo System, Bio-Rad). Membranes were blocked in 5% nonfat dried milk in PBS with 0.1% Tween-20 (PBS-T) and incubated with primary antibodies overnight at 4 °C. After three washes with PBS-T, secondary horseradish peroxidase-conjugated antibodies were added for 1 h at room temperature. Following three more washes with PBS-T, Immobilon HRP substrate (Millipore Sigma) was added, and autoradiography film (Hyblot, Thomas Scientific) was exposed and developed. For analyzing total cellular extracts, cells were lysed with SDS/PAGE sample loading buffer and sonicated using a probe sonicator (Fisher Scientific) to shear genomic DNA.

### ORF10 Affinity Purification/Mass Spectrometry.

#### First ORF10 purification—pLVX.

HEK-293T cells were transiently transfected using polyethylenimine. Forty-eight hours after transfection, cells were incubated with MG132 for 3 h before collection. Cells were lysed in 50 mM Tris pH 8.0, 10% glycerol, 150 mM NaCl, 50 mM NaF, 1 mM (ethylenedinitrilo)tetraacetic acid (EDTA), and 0.1% Nonidet P-40 with protease (cOmplete ULTRA, Roche) and phosphatase inhibitors (PhosSTOP, Roche). After centrifugation at 15,000 × *g* for 15 min, lysates were purified with Streptavidin-conjugated magnetic beads (IBA #2-4090-002). Elution of the purified proteins was carried out with PBS containing 1% SDS and incubation at 95 °C for 10 min.

#### Second ORF10 purification—pcDNA3.

HEK-293T cells were transiently transfected using polyethylenimine. Twenty-four hours after transfection, cells were collected and lysed (25 mM Tris pH 7.4, 150 mM NaCl, 10% glycerol, 0.3% Triton-X-100, and 0.1% Nonidet P-40) supplemented with protease and phosphatase inhibitors for 30 min. Lysates were centrifuged at 15,000 × *g* for 15 min; the supernatant was used for purification with Streptavidin-conjugated magnetic beads (IBA #2-4090-002). Elution of the purified proteins was carried out with elution buffer (25 mM Tris-Cl, 150 mM NaCl, 1 mM EDTA, and 50 mM Biotin) and incubated with constant rotation at room temperature for 15 min.

#### Sample preparation.

Affinity-purified pLVX samples were separated on SDS/PAGE gels, and pcDNA3 samples were prepped using S-Trap microcolumns (Protifi) according to the manufacturer’s instructions. The samples were reduced, alkylated, digested with trypsin, and desalted as previously described (26, 27). An aliquot of each sample was loaded onto an Acclaim PepMap trap column (75 μm in diameter [ID] × 2 cm, 3-μm bead size, 100-Å pore size) in line with an EASY-Spray PepMap analytical column (75 μm ID × 50 cm C18, 2-μm bead size, 100-Å pore size) using the autosampler of an EASY-nLC 1000 high-performance liquid chromatography (HPLC) (Thermo Fisher) and solvent A (2% acetonitrile, 0.5% acetic acid). The peptides were eluted into the Orbitrap QExactive (Thermo Fisher Scientific) or the Orbitrap Eclipse Mass Spectrometer (Thermo Fisher Scientific) using the following gradient: 5 to 35% solvent B (80% acetonitrile, 0.5% acetic acid) over 60 min, followed by an increase from 35 to 45% solvent B over 10 min, followed by an increase of 45 to 100% solvent B in 10 min.

#### Mass spectrometry settings.

The pLVX samples were acquired on the Orbitrap Eclipse using the following parameters: full mass spectrometry (MS) spectra resolution of 240,000 (at *m/z* 200), an automatic gain control (AGC) target of 1e6, maximum ion time of 50 ms, and scan range from 400 *m/z* to 1,500 *m/z*. Following each full MS scan, low-resolution MS/MS spectra were acquired for a 1-s duty cycle. The MS/MS spectra were collected in the ion trap in rapid scan mode, with an AGC target of 2e4, maximum ion time of 18 ms, one microscan, 2-*m/z* isolation window, normalized collision energy (NCE) of 27, and a dynamic exclusion of 30 s. The pcDNA3 samples were acquired on the Orbitrap QExactive using the following parameters: full MS spectra resolution of 70,000 (at *m/z* 400), AGC target of 1e6, with a maximum ion time of 120 ms, and scan range from 400 *m/z* to 1,500 *m/z*. Following each full MS scan, 20 data-dependent MS/MS spectra were acquired using a resolution of 17,500, an AGC target of 5e4, maximum ion time of 120 ms, one microscan, 2-*m/z* isolation window, fixed first mass of 150 *m/z*, dynamic exclusion of 30 s, and NCE of 27.

#### Data analysis.

All acquired MS/MS spectra were searched against a UniProt human database using Sequest within Proteome Discoverer 1.4 (Thermo Scientific). The search parameters were as follows: precursor mass tolerance ± 10 ppm, fragment mass tolerance ±0.4 Da for ion trap MS/MS and ±0.02 Da for Orbitrap MS/MS, digestion parameters allowing trypsin 2 missed cleavages, fixed modification of carbamidomethyl on cysteine, variable modification of oxidation on methionine, and variable modification of deamidation on glutamine and asparagine. Peptides were filtered to better than 1% FDR using a target−decoy database strategy, and proteins require at least two unique peptides to be reported. To identify potential binding partners of ORF10, the data were further analyzed using the SAINT algorithm (11). Here, 13 control affinity purifications from previous HEK-293T experiments acquired in-house were included as negative controls for comparison.

### TMT Mass Spectrometry.

#### Lysis and digestion.

Frozen 293T cell pellets (biological triplicate) from 10-cm plates were lysed in 8M Urea pH 8.5 in 200 mM 3-[4-(2-Hydroxyethyl)piperazin-1-yl]propane-1-sulfonic acid (EPPS), supplemented with a protease inhibitor tablet (Roche). Following vortexing to disrupt the pellet, samples were incubated on ice for 20 min to achieve lysis, then incubated with benzonase for 15 min at room temperature to disrupt chromatin. Lysates were cleared by centrifugation at 21,000 × *g* at 4 °C for 20 min, and supernatant was transferred to a new tube for determination of protein concentration via a bicinchoninic acid assay (ThermoFisher Scientific). Proteins were reduced with 5 mM tris(2-carboxyethyl)phosphine for 15 min at room temperature, followed by alkylation with 15 mM iodoacetamide for 30 min at room temperature (protected from light). Samples were quenched with 5 mM DTT for 15 min protected from light. Then 100 μg of each sample was subjected to methanol chloroform precipitation, and protein pellets were dried at room temperature, then digested with LysC protease (100:1 protein: protease ratio) in 200 mM EPPS at 37 °C overnight. Trypsin protease was added at a 100:1 protein:protease ratio, and digestion continued for 6 h at 37 °C.

#### TMT labeling.

Following trypsin digest, samples were cooled to room temperature, and anhydrous acetonitrile (ACN) was added to each sample to a final concentration of 30% (vol/vol). Isobaric labeling of peptides was performed using TMT nine-plex reagents (Thermo Fisher Scientific). TMT reagents (5 mg) were dissolved in 256 µL of anhydrous ACN, and 10 μL of each TMT reagent was added to 50 μg of digested peptides (half of digest volume). Following incubation at room temperature for 1 h, samples were frozen at −80 °C. Samples were thawed the following day and pooled across all nine TMT-labeled channels. The pooled sample was vacuum centrifuged to near dryness and subjected to a C18 solid-phase extraction column with a capacity of 100 mg (Sep-Pak, Waters).

#### Off-line basic pH reversed-phase fractionation.

The desalted peptide sample was fractionated using basic pH reversed-phase HPLC (28) and an Agilent 1200 pump equipped with a degasser and an ultraviolet detector (set at 220- and 280-nm wavelength). Peptides were subjected to a 50-min linear gradient from 5 to 35% acetonitrile in 10 mM ammonium bicarbonate pH 8 at a flow rate of 0.6 mL/min over an Agilent ZORBAX 300Extend C18 column (3.5-μm particles, 4.6 mm ID and 250 mm in length). The peptide mixture was fractionated into a total of 96 fractions, which were consolidated into 24 fractions. Final samples were prepared for every other fraction (12 samples in total). Fractions were acidified with 5% formic acid and vacuum centrifuged to near dryness, followed by desalting via StageTip. Dried samples were reconstituted in 5% acetonitrile/5% formic acid for LC-MS/MS analysis.

#### Data acquisition.

Mass spectrometric data were collected on an Orbitrap Eclipse mass spectrometer coupled to a Proxeon NanoLC-1200 ultra-high-performance liquid chromatography (UHPLC). The 100-μm capillary column was packed with 35 cm of Accucore 150 resin (2.6 μm, 150 Å; ThermoFisher Scientific). The scan sequence began with an MS1 spectrum (Orbitrap analysis, resolution 120,000, 350 to 1,400 Th, AGC target 5 × 10^5^, maximum injection time 100 ms). Data were acquired ∼120 min per fraction. MS2 analysis consisted of collision-induced dissociation, quadrupole ion trap analysis, AGC 2 × 10^4^, NCE 35, q value 0.25, maximum injection time 120 ms), isolation window at 0.5 Th, and TopSpeed set at 3 s. For field asymmetric ion mobility spectrometry, the dispersion voltage was set at 5,000 V; the compensation voltages used were −40, −60, and −80 V; and the TopSpeed parameter was set at 1 s.

#### Data processing.

A compendium of in-house software was used to convert files to mzXML format, as well as to correct monoisotopic *m/z* measurements and erroneous charge state assignments. Assignment of MS/MS spectra was performed using the Comet algorithm (v.2018.01 rev.2). Database searching included all entries from the human UniProt Database (August 2019), supplemented with all SARS-CoV-2 protein entries. Searches were performed using a 50-ppm precursor ion tolerance, and the product ion tolerance was set to 0.9 Da. Trypsin protease specificity was required, allowing up to two missed cleavages. TMT tags on peptide N termini/lysine residues (+229.1629 Da) and carbamidomethylation of cysteine residues (+57.0215 Da) were set as static modifications, while methionine oxidation (+15.9949 Da) was set as a variable modification. Peptide spectrum matches (PSMs) were adjusted to a 1% FDR (29, 30). PSM filtering was performed as previously described (30) using an in-house linear discrimination analysis algorithm considering the following parameters: XCorr, peptide ion mass accuracy, charge state, peptide length, and missed cleavages. For TMT-based reporter ion quantitation, the signal-to-noise ratio (S:N) for each TMT channel was extracted, and the closest matching centroid to the expected mass of the TMT reporter ion was identified. PSMs were identified, quantified, collapsed to a peptide FDR of 1%, and then collapsed further to a final protein-level FDR of 1%. Protein assembly was guided by principles of parsimony to produce the smallest set of proteins necessary to account for all of the observed peptides, using an in-house protein assembly algorithm. Peptide intensities were quantified by summing reporter ion counts across all matching PSMs using in-house software, as described previously (31, 32). A 0.003 Th window around the theoretical *m/z* of each reporter ion was scanned, and the maximum intensity nearest to the theoretical *m/z* was used. PSMs with MS3 spectra with TMT reporter summed S:N of <100 were excluded from quantitation, and isolation specificity of >0.7 was required (31, 33). Protein quantitation values were exported, and student’s *t* tests were performed in R (v4.0.2). FDR scores were calculated using the p.adjust function, and plots were generated in R as well.

### Cultivation of SARS-CoV-2.

SARS-CoV-2 (USA_WA1/2020 strain) was provided by the University of Texas Medical Branch Arbovirus Reference Collection and cultivated on VeroE6 cells (ATCC). Culture supernatants were collected 3 d postinfection and clarified by centrifugation. Titer was calculated by serially diluting virus on VeroE6 cells and performing a focus-forming unit assay for virus infection by immunofluorescence detection of virus nucleoprotein. All SARS-CoV-2 experiments were performed under biosafety level 4 conditions in the National Emerging Infectious Disease Laboratories BSL-4 suite at Boston University.

### Coronavirus Infection in KO Cell Lines.

Cells were plated onto 96-well plates coated with ECL Cell Attachment matrix (Millipore Sigma) diluted to 20 μg/mL in serum-free DMEM to enhance cell attachment. Cells were infected at an MOI of 0.4 and incubated for 48 h at 37 °C, then fixed in 10% neutral buffered formalin. Samples were assessed for infection efficiency via immunofluorescence. Samples were imaged on a Cytation 1 Cell Imaging Multi-Mode Reader (BioTek) and were evaluated using CellProfiler (34). Infection efficiencies were calculated from the percentage of viral nucleoprotein positive cells in each sample.

### Immunofluorescence.

Samples were permeabilized in 0.1% TritonX and blocked in 3.5% BSA. Primary antibody was diluted 1:1,000 in BSA and incubated overnight at 4 °C. Samples were washed twice in PBS and incubated with secondary antibodies diluted 1:1,500 in BSA for 2 h at room temperature. Cell nuclei were stained with Hoechst 33342 (Invitrogen).

## Supplementary Material

Supplementary File

Supplementary File

Supplementary File

## Data Availability

All study data are included in the article and Datasets S1−S3.
